# Heterogeneous catalyst design by generative adversarial network and first-principles based microkinetics

**DOI:** 10.1038/s41598-022-15586-9

**Published:** 2022-07-08

**Authors:** Atsushi Ishikawa

**Affiliations:** Center for Green Research on Energy and Environmental Materials (GREEN), National Institute for Materials Science (NIMS), 1-1 Namiki, Tsukuba, Ibaraki 305-0044 Japan

**Keywords:** Catalysis, Physical chemistry, Theoretical chemistry

## Abstract

Microkinetic analysis based on density functional theory (DFT) was combined with a generative adversarial network (GAN) to enable the artificial proposal of heterogeneous catalysts based on the DFT-calculated dataset. The approach was applied to the NH_3_ formation reaction on Rh−Ru alloy surfaces as an example. The NH_3_ formation turnover frequency (TOF) was calculated by DFT-based microkinetics. Six elementary reactions, namely, N_2_ dissociation, H_2_ dissociation, NH_*x*_ (*x* = 1–3) formation, and NH_3_ desorption, were explicitly considered, and their reaction energies were evaluated by DFT calculations. Based on the TOF values and atomic compositions, new alloy surfaces were generated using the GAN. This approach successfully generated the surfaces that were not included in the initial dataset but exhibited higher TOF values. The N_2_ dissociation reaction was more exothermic for the generated surfaces, leading to higher TOF. The present study demonstrates that the automatic improvement of catalyst materials is possible using DFT calculations and GAN sample generation.

## Introduction

Catalysts play a crucial role in energy and environmental science, and its performance is often evaluated by the reaction rate or turnover frequency (TOF). Researchers have devoted great efforts to find new and more active catalysts. It is well known that the reaction rate is governed by several factors: the activation energies, number of active sites (or the catalyst surface area), sticking coefficients on the surface, etc. Unfortunately, accurate measurement of such quantities requires both special expertise and considerable effort; therefore, detailed kinetic profiles have only been clarified for limited cases.

In the last few decades, theoretical simulation and computational methods have become feasible alternatives for the evaluation of reaction kinetics. For example, ab initio or first-principles calculation are popular because they provide atomic-scale information without requiring experimental data. For example, these approaches can be helpful in identifying the active site on the catalyst surface, which is a fundamental issue in catalysis. Owing to recent developments in algorithms and computational resources, such atomic-scale simulations, especially those using density functional theory (DFT), are being widely employed.

While computational methods are useful for studying given real or proposed materials, they cannot automatically suggest new ones. Recently, the application of machine learning to computational chemistry was found to afford a computation-based material proposal^1^. Several promising examples have been reported in catalysis^2–5^. Among the several possibilities of combining computational chemistry with machine learning, the present author considers the so-called generative model a particularly important machine learning algorithm because it enables “extrapolative” proposal in the material or configuration space; hence, the search is not confined within a given dataset. As an example of a generative model, the generative adversarial network (GAN) is widely used, especially in artificial image generation^6,7^. Several groups have reported the application of a GAN to material science; Kim et al*.* used it to discover new zeolite systems^8^, and several groups also used it to artificially generate crystal structures with desirable properties^9,10^.

A catalytic reaction often involves several species and elementary reactions. Microkinetic analysis explicitly treats a set of elementary reactions. Therefore, it is often more accurate than the kinetic analysis based on the global rate expression^11^. Currently, DFT-based microkinetics is widely used in catalysis research because it is a powerful tool that allows the calculation of kinetic information, such as the reaction energy and activation barrier, can be calculated by DFT^11–15^. Considering this, a combination of DFT calculations, microkinetics, and catalytic material generation from generative models could be a promising approach for the rational design of catalysts.

The present paper describes a new approach based on DFT calculations and sample generation by a GAN for heterogeneous catalyst searching. The generation procedure is extrapolative, because the proposed catalytic material need not be included in the initially prepared dataset. Here, the GAN part aims to generate materials with a high TOF for the target catalytic reaction, where the TOF is calculated using DFT-based microkinetics. The ammonia (NH_3_) synthesis reaction, which is also known as the Haber−Bosch process, is considered in this study as a representative heterogeneous catalytic reaction^16–18^. Below, details of the DFT-GAN procedure are described in the Methods section, and its performance is discussed in the Results and Discussion section.

## Methods

### Models and details of DFT calculation

**Figure 1:**
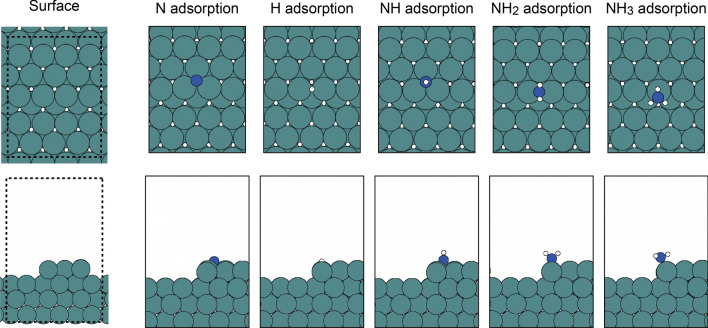
Typical slab model for the stepped surface. The adsorption sites of the N, H, NH, NH_2_, and NH_3_ species are also provided. The upper and lower panels show the top and side views. The dotted line in the bare surface model indicates the boundary of the supercell. The metal, N, and H atoms are represented by green, blue, and white spheres, respectively.

For the target model, the catalytic synthesis of NH_3_ was assumed to occur on a Rh−Ru bimetallic alloy surface. The Ru stepped surface was constructed first, and the bimetallic alloys were constructed by replacing Ru atoms with Rh atoms. Stepped metal surfaces were considered because NH_3_ formation is known to occur on these types of surfaces^17,19^. The positions of the Ru atoms replaced by Rh atoms were randomly selected in the original dataset, while during the DFT-GAN iterations the positions of the Rh atoms were determined by the GAN; the details will be discussed later. The original dataset included 100 metal surfaces. The metal surfaces were modeled by repeated slabs, and the stepped surface was modeled by removing half of the atoms in the top layer. Each slab consisted of a (6 × 4) supercell in the lateral direction, with four atomic layers in the z-direction. Consequently, 84 atoms were included in the model. The typical structure of the surface model is shown in Fig. 1. The adsorption positions of N, H, NH, and NH_2_ were assumed to be the fcc three-fold hollow sites, and atop adsorption was assumed for NH_2_ and NH_3_, as these positions are the most stable adsorption sites on the Ru-stepped surface^20^. These adsorption sites are also shown in Fig. 1.

The BEEF-vdW exchange–correlation functional was used in the DFT calculations because it provides an accurate description of the van der Waals interaction^21^. The core electrons were represented by the projector-augmented wave (PAW) potentials^22^, and the valence electrons were expanded with plane waves up to a cutoff energy of 400 eV. Spin polarization was included throughout, and no symmetry constraint was imposed on the geometries. A Gaussian scheme was used in the smearing of the electron occupation close to the Fermi level. The convergence thresholds for the electronic and geometry optimizations were set to 1.0 × 10^−4^ eV and 1.0 × 10^−1^ eV/Å in energy and force, respectively.

The geometries of the surfaces generated by the GAN were optimized using DFT, with the same computational condition as the initial dataset. A vacuum layer of ~ 12 Å was placed between the slabs, and dipole correction was applied in the z-direction to cancel the artificial interactions between slabs. As the substitution of Rh for Ru causes some distortion in the unit cell, the unit cell was optimized for all surfaces; the BEEF-vdW functional was also used for this purpose, and the convergence threshold for the unit cell optimization was set to 1.0 × 10^−4^ eV in energy change. An orthorhombic unit cell was used in all cases. All DFT calculations were performed using the Vienna ab initio simulation package (VASP), version 5.4.4^23,24^.

### Elementary reactions and rate of NH_3_ formation

The overall reaction for the synthesis of NH_3_ is represented by:

1 $${\text{N}}_{{2}} \,\, + \,\,3{\text{H}}_{{2}} \to 2{\text{NH}}_{{3}}$$

which is generally considered to include the following six elementary reactions^25^.

2 $${\text{N}}_{{2}} \,\, + \,\,2{*} \to 2{\text{N*}}$$

3 $${\text{H}}_{{2}} \,\, + \,\,2{*} \rightleftharpoons 2{\text{H*}}$$

4 $${\text{N*}}\,\, + \,\,{\text{H*}} \rightleftarrows {\text{NH*}}\,\,{ + }\,\,{*}$$

5 $${\text{NH*}}\,\, + \,\,{\text{H*}} \rightleftarrows {\text{NH}}_{{2}} {*}\,\,{ + }\,\,{*}$$

6 $${\text{NH}}_{{2}} {*}\,\, + \,\,{\text{H*}} \rightleftarrows {\text{NH}}_{{3}} {*}\,\,{ + }\,\,{*}$$

7 $${\text{NH}}_{{3}} {*} \rightleftarrows {\text{NH}}_{{3}} \,\,{ + }\,\,{*}$$

where an asterisk (*) denotes a vacant active site on the metal surface, and the species with asterisks are the adsorbed species. The reaction energies in Eqs. (2)–(7) were determined from the total energy calculated by DFT, i.e., the sum of the electronic and nuclear repulsion energies.

Previous experimental and theoretical research suggests that the rate-determining step (RDS) is Eq. (2), namely, the dissociative adsorption of N_2_^17,26^. Based on this assumption, the present study employed the simple microkinetic model, called the Langmuir−Hinshelwood−Hougen−Watson kinetic model, wherein the RDS was assumed to be Eq. (2) irrespective of temperature and pressure changes. In this case, the fractional surface coverage of the adsorbed species *i* (*θ*_*i*_) is written as

8 $$\begin{gathered} \theta_{{\text{N}}} = \frac{{p_{{{\text{NH}}_{{3}} }} }}{{K_{3}^{3/2} p_{{{\text{H}}_{2} }}^{3/2} K_{4} K_{5} K_{6} K_{7} }}\theta_{{{\text{vac}}}} \hfill \\ \theta_{{\text{H}}} = \sqrt {K_{3} p_{{{\text{H}}_{{2}} }} } \theta_{{{\text{vac}}}} \hfill \\ \theta_{{{\text{NH}}}} = \frac{{p_{{{\text{NH}}_{{3}} }} }}{{K_{3} p_{{{\text{H}}_{2} }} K_{5} K_{6} K_{7} }}\theta_{{{\text{vac}}}} \hfill \\ \theta_{{{\text{NH}}_{{2}} }} = \frac{{p_{{{\text{NH}}_{{3}} }} }}{{\sqrt {K_{3} p_{{{\text{H}}_{2} }} } K_{6} K_{7} }}\theta_{{{\text{vac}}}} \hfill \\ \theta_{{{\text{NH}}_{3} }} = \frac{{p_{{{\text{NH}}_{{3}} }} }}{{K_{7} }}\theta_{{{\text{vac}}}} \hfill \\ \theta_{{{\text{vac}}}} = \frac{1}{{1 + \frac{{p_{{{\text{NH}}_{{3}} }} }}{{K_{3}^{3/2} p_{{{\text{H}}_{2} }}^{3/2} K_{4} K_{5} K_{6} K_{7} }} + \sqrt {K_{3} p_{{{\text{H}}_{{2}} }} } + \frac{{p_{{{\text{NH}}_{{3}} }} }}{{K_{3} p_{{{\text{H}}_{2} }} K_{5} K_{6} K_{7} }} + \frac{{p_{{{\text{NH}}_{{3}} }} }}{{\sqrt {K_{3} p_{{{\text{H}}_{2} }} } K_{6} K_{7} }} + \frac{{p_{{{\text{NH}}_{{3}} }} }}{{K_{7} }}}} \hfill \\ \end{gathered}$$

where *p*_*i*_ is the partial pressure of NH_3_ or H_2_, and *K*_*i*_ is the equilibrium constant of Eqs. (2)–(7)^25^. Thus, the total reaction rate is written as

9 $$R = k \cdot p_{{{\text{N}}_{{2}} }} \theta_{{{\text{vac}}}} \left( {1 - \frac{1}{{K_{2} K_{3}^{3} K_{4}^{2} K_{5}^{2} K_{6}^{2} K_{7}^{2} }}\frac{{p_{{{\text{NH}}_{{3}} }}^{2} }}{{p_{{{\text{N}}_{{2}} }} p_{{{\text{H}}_{{2}} }}^{3} }}} \right)$$

where *k* is the rate constant of the RDS (Eq. (2)) calculated using the Arrhenius equation:

10 $$k = A\exp \left( { - \frac{{E_{a} }}{{R_{{{\text{gas}}}} T}}} \right).$$

*E*_a_ is the activation energy of Eq. (2), *A* is the pre-exponential factor with the value of 0.241 s^−1^ given by Logadottir et al*.*^20^, *R*_gas_ is the universal gas constant, and *T* is the temperature. For the zero-point energies and thermal correction terms, experimental values from the NIST webbook were used^27^.

Although it is possible to evaluate *E*_a_ with DFT by locating the transition state, this process requires considerable computational effort. Instead, this study evaluated *E*_a_ using the linear free energy relationship (or the Brønsted−Evans−Polanyi principle), in which *E*_a_ is expressed as a linear function of Δ*E* as

11 $$E_{a} = \alpha \Delta E + \beta .$$

The values of *α* = 0.87 and *β* = 1.34 for the stepped metal surface were taken from the literature^28^. The calculation was carried out at *T* = 700 K and a total pressure of 100 bar. Stoichiometric quantities of N_2_ and H_2_ were used in the inlet gas, i.e., *p*_N2_ : *p*_H2_ = 1 : 3 and the conversion of N_2_ was set to 10%. It has been previously demonstrated by Honkala et al. that the adsorbate−adsorbate interaction has some impact on the kinetics on the NH_3_ formation^17^. However, these interactions are not considered in the present system, and hence the TOF values should be considered as representing the semi-quantitative level.

### Details of the GAN

**Figure 2:**
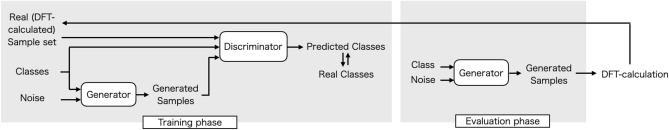
Structure of the DFT-GAN procedure used in this study. In the training phase, the generator and discriminator are trained using DFT-calculated data. Then, new samples are generated in the evaluation phase. This training−evaluation sequence consists of one iteration. After the newly generated samples are evaluated by DFT calculations, they are added to the original dataset for use in the subsequent iteration.

Similar to the original and several extended versions of the GAN, the entire system here consists of the generator (G) and discriminator (D) networks, the structures of which are shown in Fig. 2. In the present case, fake samples with a high NH_3_ formation rate are desirable, and they can be generated using G. Therefore, the conditional GAN (CGAN) was applied because it enables the generation of fake samples corresponding to a given label^7^. The metal surface was encoded by a one-dimensional string array consisting of Rh or Ru. Then the string is converted to the one-dimensional vector of either 0 or 1 value. This vector and the DFT-calculated TOF value are used as the descriptor and target values, respectively. These alloys and their DFT-calculated TOF values were used together for learning. The mean-squared error was used for the loss functions of the D and G parts^29^. Because the fake samples are expressed as a one-dimensional vector of the transition metal elements and the initial atomic positions, DFT calculations can be carried out for these samples. This also implies that their NH_3_ formation rates could be evaluated with the same accuracy as the original dataset. The DFT calculation results for the generated samples were added to the original dataset, and the augmented dataset was used for the iterative training of the GAN. The specific steps are as follows:

1. DFT calculations are performed to obtain the *E*_a_ and Δ*E* values for the elementary reactions of Eqs. (2)–(7). This is done for all samples in the dataset.
2. The TOF for NH_3_ formation is calculated according to Eq. (9) using the DFT-calculated Δ*E* and *E*_a_ values.
3. The calculated samples are sorted according to the DFT values and are grouped into several classes (*n*) according to the NH_3_ formation rate. Here, the number of groups is set to five, and the group with the highest TOF is labeled as *n* = 1.
4. Networks D and G are trained with the dataset using the backpropagation scheme.
5. G generates fake samples for *n* = 1. Any generated surface that overlaps with the existing sample set is removed.
6. DFT calculations are performed for the newly generated samples. The results are added to the dataset for use in the subsequent iteration.

It should be noted that the size of the dataset increases with increasing number of iterations. This feature is favorable in terms of training neural networks, as a larger number of samples can be used in the training. Several studies have shown that such iterative training of the GAN is effective^30,31^.

The present form of the target function only considered the reactivity. The stability of the surface is not taken into account, although it could be important for practical applications. The surface stability may be evaluated using the DFT method from the surface energy or bulk energy. Because there is no limitation in the target function for the present approach, it is possible to employ the surface stability or a mixed function of the stability and the TOF as the target function. The detailed examination of such a target function and its effect on the generated samples would be a suitable future study, however, have not been considered in the current study.

When training D and G, the loss function was set to the mean-squared error in both cases, and the ADAM optimizer was used. The learning rate was set to 1.0 × 10^−3^, and the parameters *β*_1_ and *β*_2_ were set to 0.5 and 0.999, respectively. The dropout rate was set to 0.3, and the minibatch size was set to 20% of the sample size for each iteration. The maximum number of the training processes (i.e., the epoch) was set to 2000.

The Python library atomic simulation environment (ASE) was used to construct the model and perform the DFT calculations^32^. The GAN part was calculated using PyTorch version 1.8. These Python codes are freely available on the author’s GitHub page^33^.

## Results and discussion

Initially, 100 bimetallic alloy surfaces were generated by randomly replacing Ru atoms with Rh atoms on a surface. DFT calculations were performed on these samples to obtain the TOF values. In the following, this dataset is referred to as the “original dataset.” Then, the iterative GAN procedure described above was applied. Five iterations were performed, meaning that four generations of new samples were created in addition to the original dataset.

**Figure 3:**
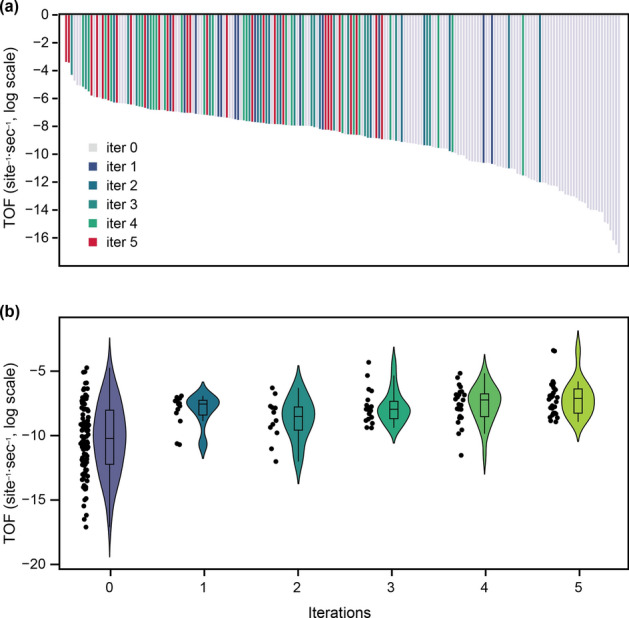
(**a**) TOF of NH_3_ formation on 224 Rh−Ru alloy surfaces. The TOF values from different DFT-GAN iterations (iter = 1–5) are coded in different colors. The iter = 0 corresponds to the original dataset. The dataset is sorted according to the DFT value, i.e., from the highest (left) to the lowest (right). The reaction temperature and total pressure were set to 700 K and 100 bar, respectively. (**b**) Box and violin plots of the TOF values for iter = 0–5. The line inside the box represents the median value. The points on the left of the violin represent the raw TOF value at each iteration.

Figure 3a plots the TOFs of the metal surfaces on a logarithmic scale, and the surfaces are sorted in descending order of TOF. In the original dataset (iter = 0), the metal surfaces have a wide range of TOF values ranging from 1.0 × 10^−4^ (Rh_8_Ru_76_) to 2.3 × 10^−19^ site^−1^·s^−1^ (Rh_50_Ru_34_). This indicates a strong dependence of the TOF on the metal surface composition. The new metal surfaces generated by the first to fifth iterations of DFT-GAN (iter = 1–5) are also depicted in the figure. It can be seen that the generated surfaces tend to have relatively higher TOF values than those of the original dataset. At iter = 3, Rh_4_Ru_80_ is generated, and it has a TOF value of 3.1 × 10^−4^ site^−1^·s^−1^; this value is higher than the maximum TOF in the original dataset. At iter = 5, the optimal TOF value (1.1 × 10^−3^ site^−1^·s^−1^) is obtained with Rh_8_Ru_76_; this TOF value is more than ten times larger than the highest value in the original dataset. These findings show that the GAN successfully generated a metal surface with high catalytic performance in an extrapolative manner. The TOF values at each iteration are summarized in the so-called violin plot in Fig. 3b. The violin-shaped curves show the probability density of the TOF values, and the boxes inside the individual curves indicate the quartiles. The plot shows that the original dataset has widely distributed TOF values. The TOF distributions of the generated surfaces (iter = 1–5) are more skewed toward the high-TOF region. This trend clearly shows that the GAN is much more efficient than the random sampling, to obtain the metal surface with a high NH_3_ formation rate.

The property of the generated surface agrees with the existing chemical or physical insights. It is widely known that NH_3_ formation is much faster on a Ru surface than on a Rh surface^34^. In other words, Ru occupies a higher position in the activity volcano plot than Rh. In this study, the surfaces generated by the GAN tend to have a high proportion of Ru; the full dataset can be found in the author's GitHub repository. These results show that the neural network captures the experimental tendency of transition metal elements by learning from the TOF of NH_3_ formation calculated by the DFT, since the alloying more Ru into the Rh surface leads a higher position in the volcano plot.

**Figure 4:**
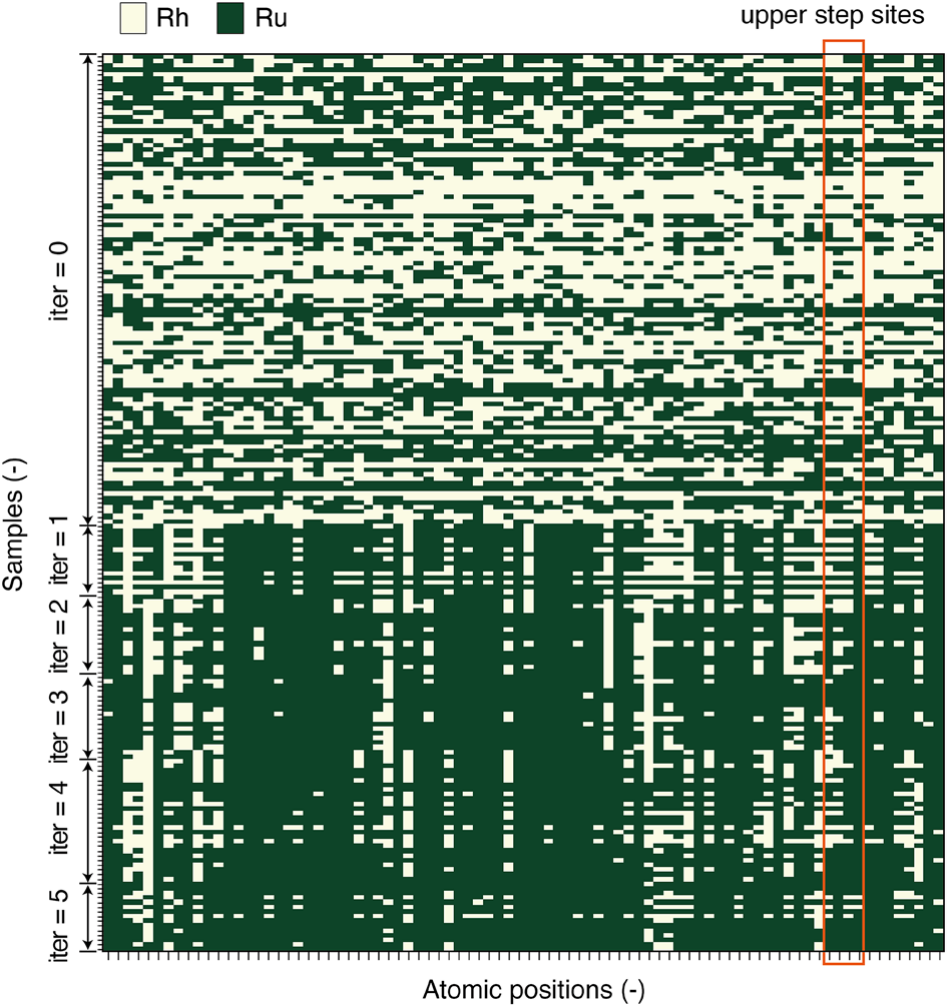
Distribution of Ru and Rh atoms in the initial (iter = 0) and the GAN-generated (iter = 1–5) surfaces. The white and green squares indicate the Rh and Ru atoms, respectively.

Further analysis was carried out to understand the atomic configuration of the GAN-generated surfaces. Figure 4 shows the distributions of the Ru and Rh atoms for all the metal surfaces. In the initial dataset (iter = 0), there is no clear tendency in the Ru and Rh distributions, as the position of Ru atoms was determined randomly. However, the proportion of Ru atoms is larger for the GAN-generated surfaces (iter = 1–5). Another significant trend is that the Ru atoms tend to occupy the step sites (marked in the figure), especially at higher iterations. These atomic positions are neighboring to the sites of the adsorbate atoms or molecules, and these neighboring atoms should have a larger impact on their adsorption energy the other atomic positions. As a result, these sites strongly affect the kinetics and thermodynamics of NH_3_ formation. The present result also indicates that the GAN successfully learned this feature and placed the Ru atoms close to the adsorption sites.

**Figure 5:**
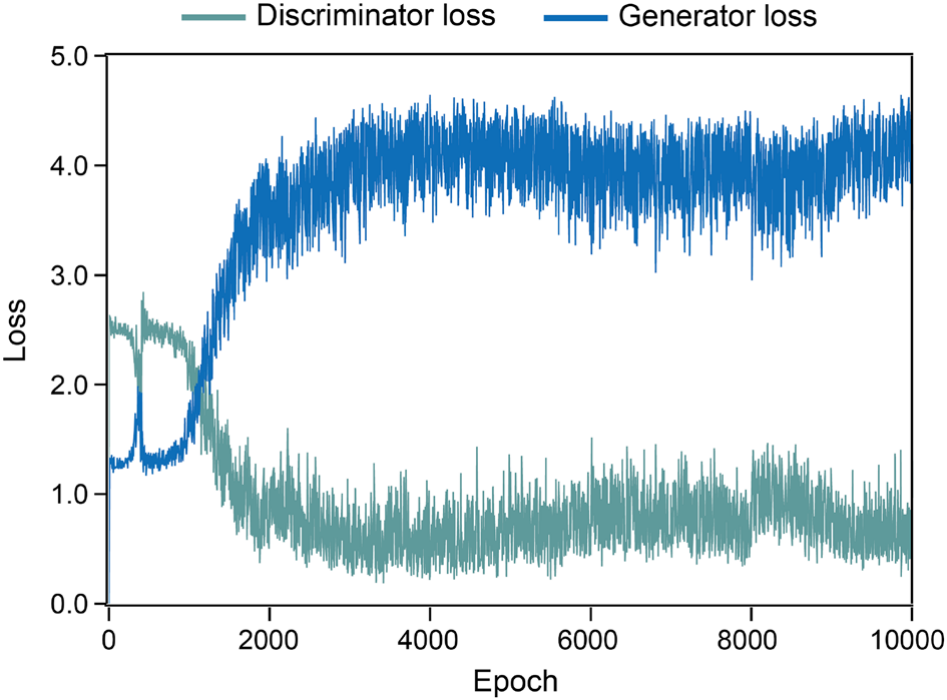
Discriminator and generator losses during the DFT-GAN procedure. Each iteration consists of 2000 epochs.

The generator loss (G-loss) and discriminator loss (D-loss) during the training process are plotted in Fig. 5. Since each iteration has 2000 epochs, there are a total of 10,000 epochs over the five iterations. In the earlier stage of training, the G-loss is smaller than the D-loss. However, after ~ 1000 epochs, the D-loss becomes lower than the G-loss, meaning that the D part of the GAN is well-trained.

**Figure 6:**
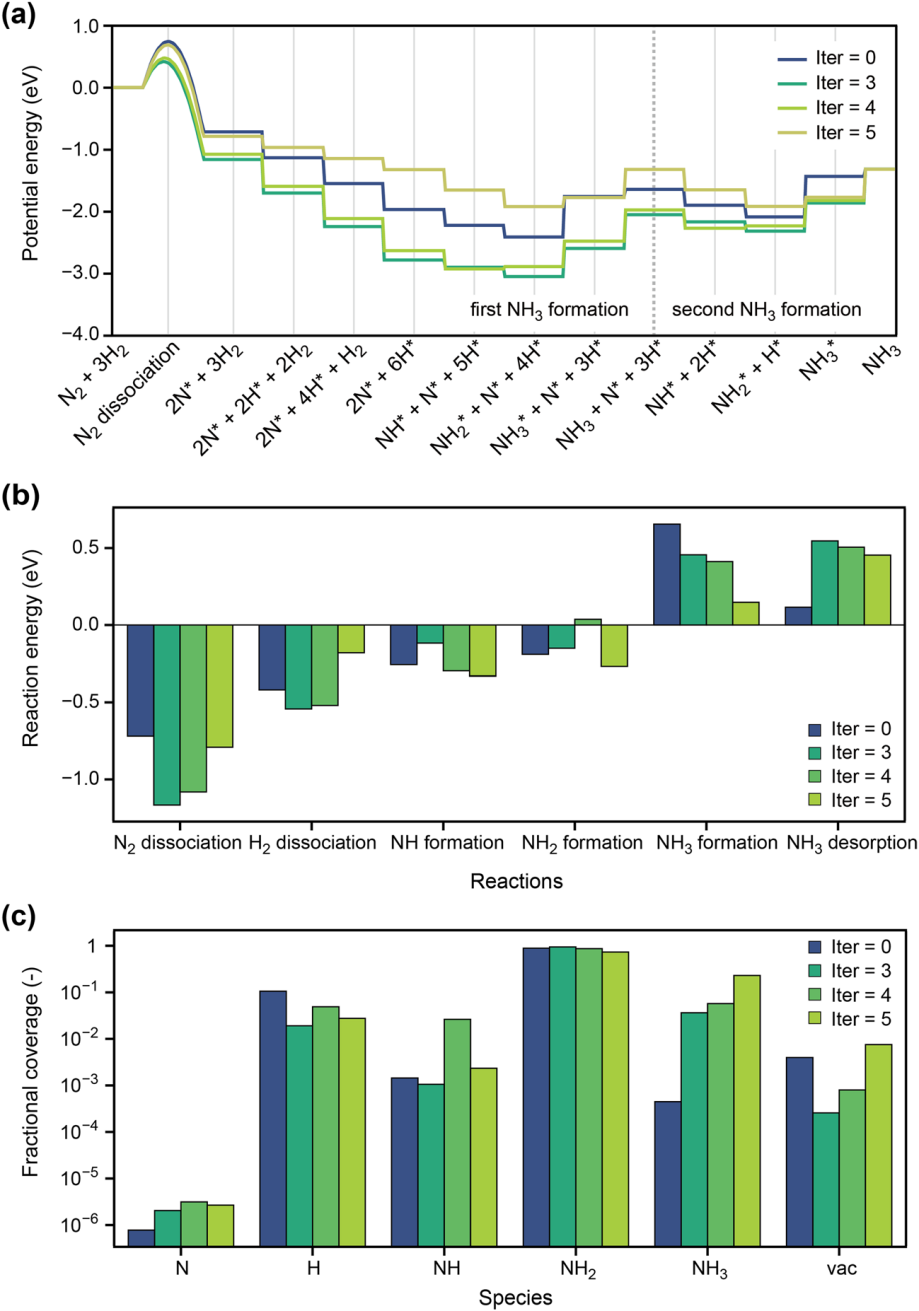
(**a**) Potential energy profile of NH_3_ formation on the Rh−﻿Ru surfaces with the highest TOF values at iter = 0, 3, 4, and 5. The activation barrier for the RDS (N_2_ dissociation) is indicated by the curves on the profiles. (**b**) Reaction energies of the six elementary steps in NH_3_ formation over the Rh−Ru surfaces. (**c**) Coverages of N, H, NH, NH_2_, NH_3_, and vacant sites (vac) on the Rh−Ru surfaces.

To understand why the generated surfaces have higher TOFs, the energetics of the NH_3_ formation reaction were analyzed. Figure 6 summarizes the potential energy curves, the reaction energies of the elementary reactions, and surface fractional coverages of the adsorbates. In this case, the Rh−Ru surfaces with the highest TOF values at iter = 0, 3, 4, and 5 are compared, and the results for all the iterations are shown in the Figures S1−S3 (Supplementary Information). The compositions of these surfaces are Rh_8_Ru_76_, Rh_4_Ru_80_, Rh_12_Ru_72_, and Rh_8_Ru_76_, respectively. The optimal TOF value (1.1 × 10^−3^ site^−1^·s^−1^) is that of Rh_8_Ru_76_, which is generated at iter = 5. This value is much higher than that of Rh_8_Ru_76_ at iter = 0 (1.0 × 10^−4^ site^−1^·s^−1^), which is the highest TOF in the original dataset (for brevity, these surfaces are denoted as Rh_8_Ru_76_-iter5 and Rh_8_Ru_76_-iter0 in the following discussion). It should also be noted that these two surfaces have the same composition, but the positions of the Rh atoms are different.

Figure 6a plots the potential energy curves for NH_3_ formation. During this process, two NH_3_ molecules are formed because the dissociation of one N_2_ molecule generates two N* (surface-adsorbed N) atoms. The plots show a deep potential energy sink at the NH_2_* + N* + 4H* state caused by the exothermic formation of NH and NH_2_ and endothermic formation of NH_3_. This energy sink is unfavorable from a thermodynamic viewpoint because the high stability of NH* and NH_2_* leads to surface poisoning. Furthermore, the lack of vacancies in the active site prohibits the next catalytic reaction; this issue will be discussed later. The potential energy curves for iter = 2–4 have lower potential energy at NH_2_* + N* + 4H*, similar to that for iter = 0. However, at iter = 5, the endothermicity of the reaction is significantly improved. This is beneficial in terms of the accessibility of the active sites to gaseous N_2_ molecules. Thus, the potential energy curves show that the GAN-generated surfaces successively improve the thermodynamic character of NH_3_ formation.

The potential energy curve of the Rh_8_Ru_76_ surface (i.e., the GAN-generated surface with the highest TOF value) is also compared with those of the Ru and Rh surfaces (Figure S4, Supplementary Information). It should be noted that the pure Ru and Rh surfaces are not included in the dataset, hence they are approximated by the RhRu_83_ and Rh_83_Ru surfaces, respectively. Interestingly, the Rh_8_Ru_76_ surface is found to be superior to the other two surfaces owing to its relatively lower *E*_a_ for the N_2_ dissociation step; as its *E*_a_ is much lower than that of the Rh surface and close to the that of the Ru surface. In addition, the hydrogen poisoning around the NH_x_ (x = 1−3) species on Rh_8_Ru_76_ is significantly reduced compared to that observed over the pure-Ru surface.

The reaction energies (Δ*E*s) of the elementary reactions (Eqs. (2)–(7)) on several surfaces are shown in Fig. 6b. The reaction energy of N_2_ dissociation (Eq. (2)) is more exothermic at iter = 2–5. For example, the Δ*E* on Rh_8_Ru_76_-iter5 is −0.79 eV, which is lower than that on Rh_8_Ru_76_-iter0 (−0.72 eV). Consequently, the activation barrier *E*_a_ becomes lower in the latter owing to the linear free energy relationship (Eq. (11)). Significantly, the GAN-generated surfaces have lower *E*_a_ at earlier iterations, while at latter iterations, it focused more on the energy sink in the potential energy profile. This feature can be understood in terms of the volcano curves: the over-stabilization of the N atom leads to a lower TOF even though the corresponding *E*_a_ is also low. The present results suggest that the GAN learned such a tendency from the data. Another notable feature is the Δ*E* for NH_3_ formation. As stated above, the facile formation of NH_3_ alleviates surface poisoning by NH_2_. The Fig. 6b shows that the value of Δ*E* for the NH_3_ formation progressively becomes less endothermic as the iteration proceeds. This suggests that the GAN improves not only the kinetics but also the thermodynamics of the NH_3_ formation.

Figure 6c compares the fractional coverages (*θ*) of the different adsorbates (N, H, NH, NH_2_, and NH_3_) and the vacant sites (i.e., the active sites) over several Rh−Ru surfaces. A notable difference is seen in the coverage of the vacant site (*θ*_vac_); for example, *θ*_vac_ is 7.5 × 10^−3^ for Rh_8_Ru_76_-iter5, but 3.9 × 10^−3^ for Rh_8_Ru_76_-iter0. The higher *θ*_vac_ for Rh_8_Ru_76_-iter5 facilitates NH_3_ formation by leaving accessible active sites for the N_2_ dissociation reaction. In accordance with its higher *θ*_vac_, Rh_8_Ru_76_-iter5 has a lower *θ*_NH2_ (0.73) than Rh_8_Ru_76_-iter0 (0.89). Furthermore, since NH_2_ is the most abundant adsorbate during NH_3_ formation, a high *θ*_NH2_ indicates NH_2_ poisoning on the surface to reduce the NH_3_ formation rate, which is known to be a serious disadvantage of Ru surfaces^35^. Thus, lowering the *θ*_NH2_ and *θ*_H_ values is desirable for the catalytic performance. The present data indicate that the GAN-generated surface has a lower *θ*_NH2_ value than those included in the original dataset.

All these results show that the proposed DFT-GAN improves the TOF of NH_3_ formation by adjusting the detailed energetics of the elementary reactions. Although the GAN was not explicitly provided with such information, training the neural networks with DFT data successfully captured these details.

## Conclusions

The present paper proposes a new approach combining computational chemistry and machine learning to generate new catalytic surfaces in an extrapolative manner. Density functional theory (DFT) is used to calculate the energies of elementary reactions on a provided set of catalytic materials. The results are fed into a generative adversarial network (GAN) to propose additional materials. Here, the approach was used to enhance the turnover frequency (TOF) of NH_3_ synthesis in the Rh−Ru bimetallic alloy surface system. The DFT-GAN iterations consist of the following six key steps. (i) DFT is used to obtain the reaction energies (Δ*E*) of the elementary reactions; this is performed for all surfaces in the initially prepared dataset. (ii) The TOF for NH_3_ formation is obtained from the Δ*E* values assuming N_2_ dissociation to be the rate-determining step, and the metal surfaces are labeled according to the TOF values. (iii) The GAN consisting of the discriminator and the generator is trained using the above DFT dataset that has metal surface information and TOF values. (v) The generator part of the GAN produces samples that are not contained in the present dataset; the conditional GAN is used here, and the generator part aims to produce surfaces with higher TOF values. (vi) DFT calculations are performed for the newly generated samples, and the results are added to the dataset.

The iterative process was started with 100 stepped alloy surfaces generated by random atomic replacement. After five iterations, Rh_8_Ru_76_ was successfully obtained as a surface not present in the original dataset. The TOF of the generated surface was more than ten times higher than the optimal TOF value in the original dataset. Overall, the samples generated in later iterations tend to have higher TOFs, indicating that the iterative DFT-GAN scheme helps train the neural networks in the GAN. Furthermore, the generated surfaces generally have a higher proportion of Ru atoms, which agrees with the experimental fact that the Ru surface is a far better catalyst than the Rh surface. The generated surfaces have higher TOF value because of (a) a lower N_2_ dissociation reaction energy (which reduces the activation energy for the rate-determining step) and (b) a lower energy of NH_3_ formation (which reduces NH_2_ coverage on the surface and alleviates NH_2_ poisoning). The present study shows that the combination of the DFT and the GAN is a promising strategy for the automatic and continuous improvement of catalyst performances.

## Supplementary Information

Supplementary Information.

## Data Availability

The datasets generated and analyzed during the current study are available in the authors' GitHub repository at https://github.com/atsushi-ishikawa.
