# Supplementary material for: Heterogeneous catalyst design by generative adversarial network and first-principles based microkinetics
Source: Sci Rep. 2022 Jul 8;12:11657. doi: 10.1038/s41598-022-15586-9 (PMC9270484; doi:10.1038/s41598-022-15586-9)
Supplement: Supplementary file 1 — Supplementary Information. [file 41598_2022_15586_MOESM1_ESM.docx]

**Heterogeneous Catalyst Design by Generative Adversarial Network and First-Principles Based Microkinetics**

**Supplementary Information**

Atsushi Ishikawa ^*^

(*E-mail: ISHIKAWA.Atsushi@nims.go.jp)

Center for Green Research on Energy and Environmental Materials (GREEN), National Institute for Materials Science (NIMS), 1-1 Namiki, Tsukuba, Ibaraki 305-0044, Japan


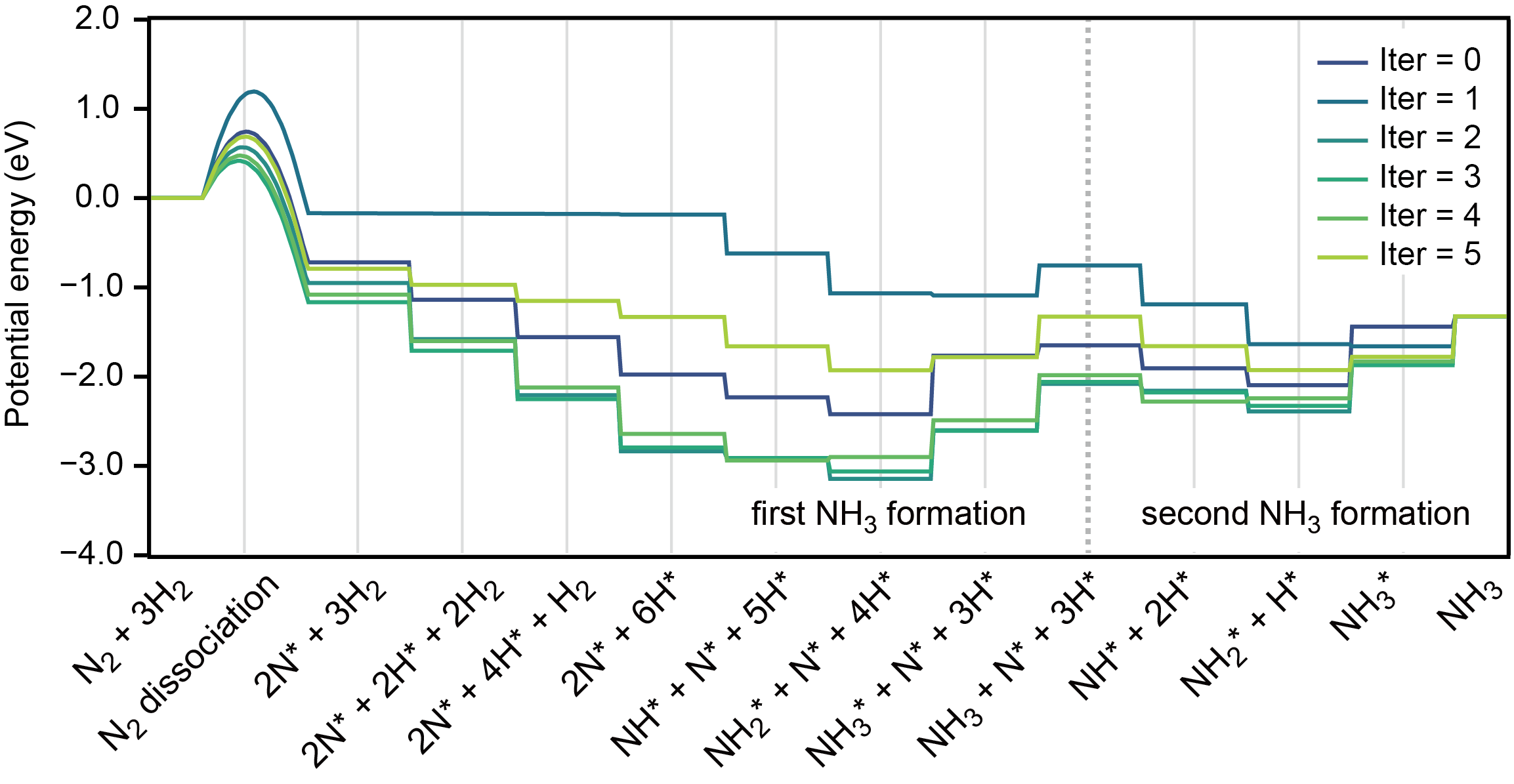


Figure S1. Potential energy profile of NH_3_ formation on the Rh-Ru surfaces with the highest TOF at iter = 0-5. The activation barrier for the RDS (N_2_ dissociation) is indicated by the curves on the profile.


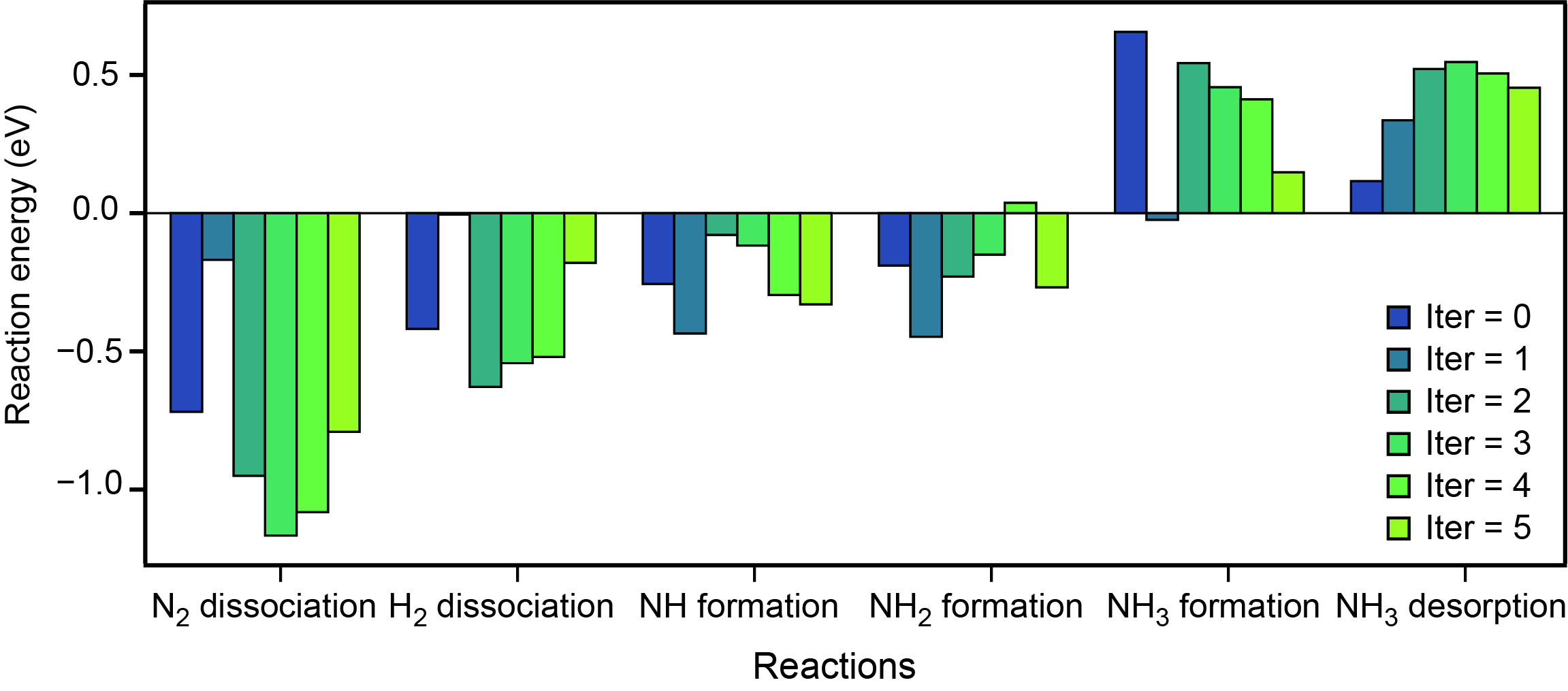


Figure S2. Reaction energies of the six elementary steps in NH_3_ formation over the Rh-Ru surfaces, at iter = 0-5.


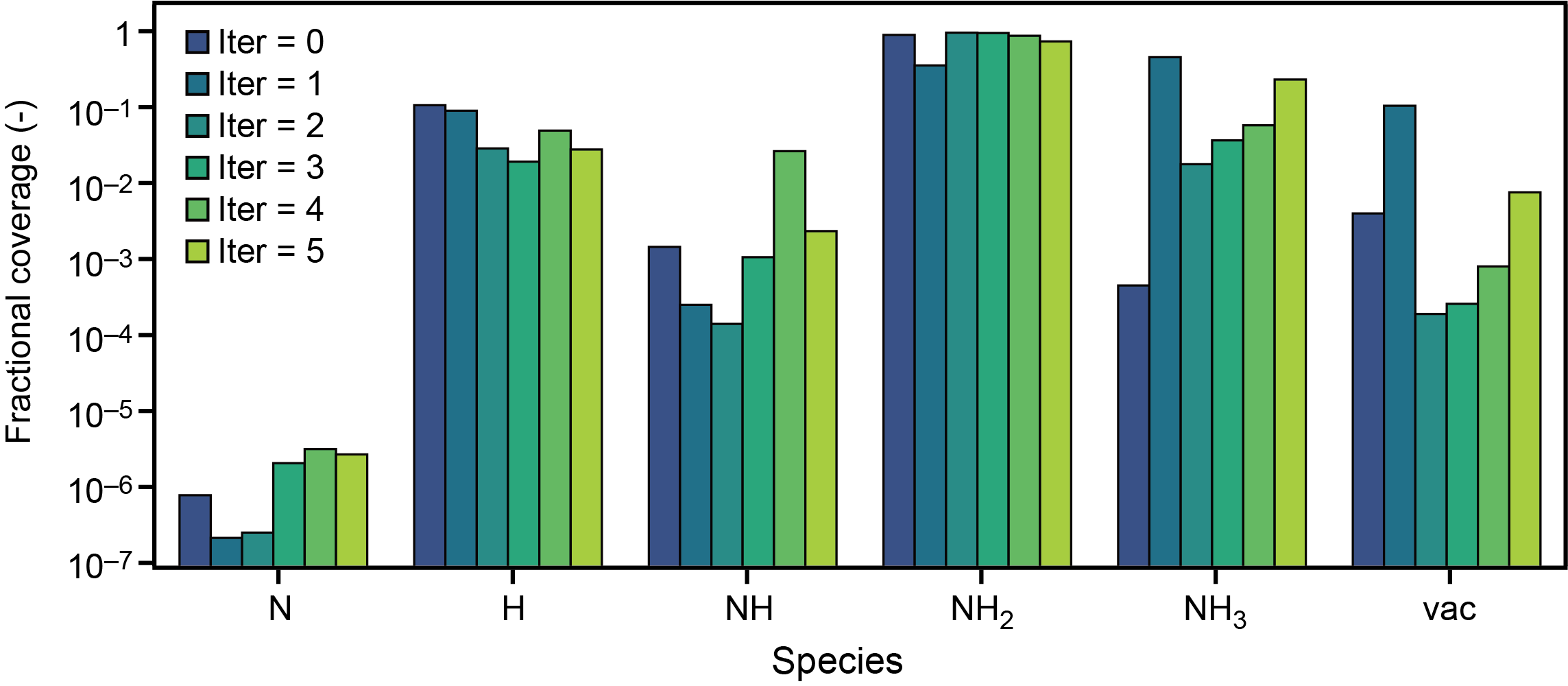


Figure S3. Coverages of N, H, NH, NH_2_, NH_3_, and vacant site (vac) on the Rh-Ru surfaces, at iter = 0-5.


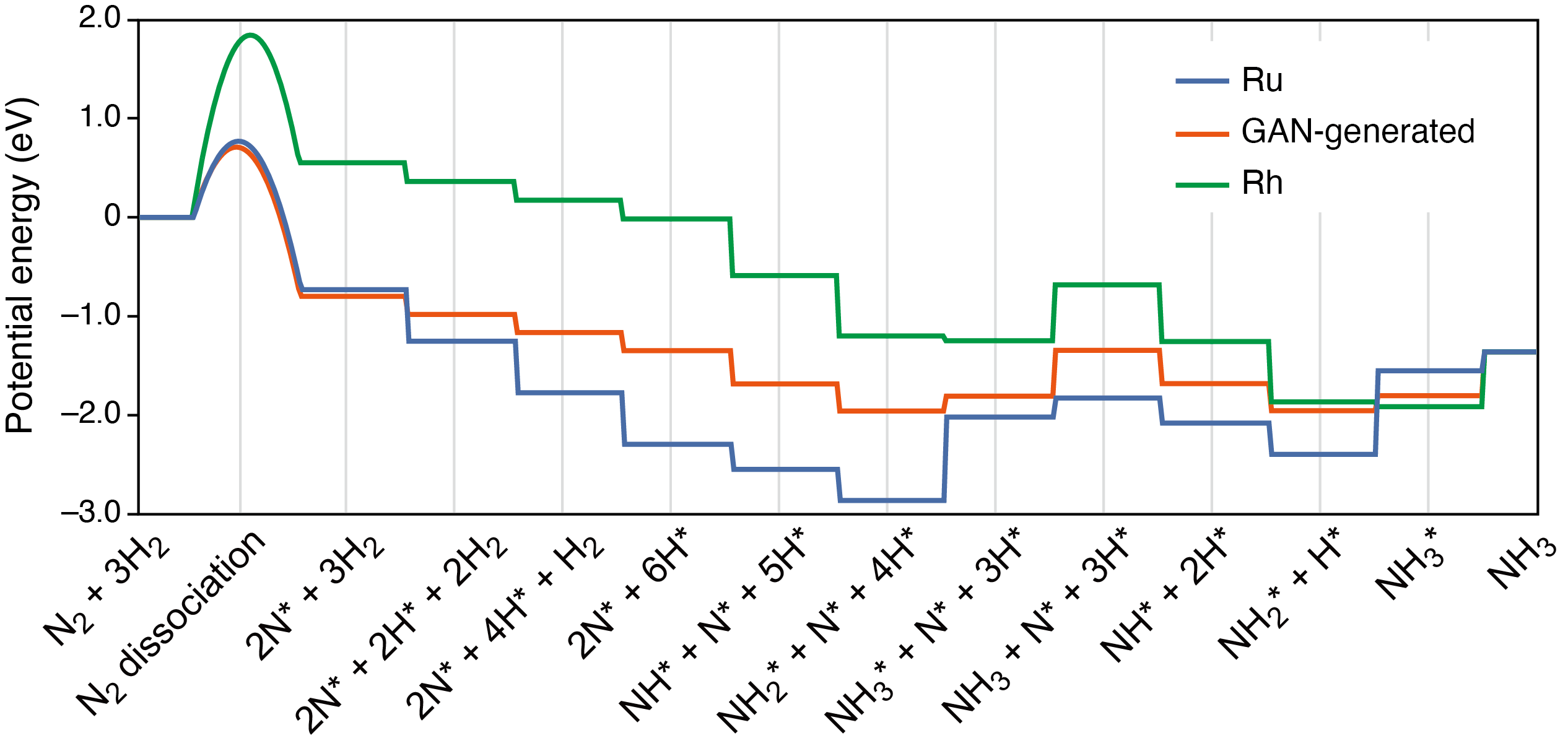


Figure S4. The potential energy curves for Ru, Rh, and the GAN-generated surface with highest TOF value (Rh_8_Ru_76_). Ru and Rh were approximated with RhRu_83_ and Rh_83_Ru surfaces, respectively, as pure Ru and Rh surfaces were not included in the dataset.
